# The role of FV *1691G>A*, FII *20210G>A* mutations and MTHFR *677C>T*; *1298A>C* and *103G>T* FXIII gene polymorphisms in pathogenesis of intraventricular hemorrhage in infants born before 32 weeks of gestation

**DOI:** 10.1007/s00381-017-3460-8

**Published:** 2017-06-03

**Authors:** Dawid Szpecht, Janusz Gadzinowski, Agnieszka Seremak-Mrozikiewicz, Grażyna Kurzawińska, Krzysztof Drews, Marta Szymankiewicz

**Affiliations:** 1 Chair and Department of Neonatology, Poznan University of Medical Sciences, Polna 33 Street, 60-535 Poznań, Poland; 2 Department of Perinatology and Women’s Diseases, Poznan University of Medical Sciences, Poznan, Poland; 3 Department of Pharmacology and Phytochemistry, Institute of Natural Fibers and Plants, Poznan, Poland

**Keywords:** Gene, Polymorphism, Intraventricular hemorrhage, Preterm newborn

## Abstract

**Background:**

Congenital thrombophilia is associated with an increased intraventricular hemorrhage (IVH) risk among newborns, but it may also play a protective role. The role of genetic polymorphisms involved in the coagulation pathway of IVH pathogenesis is probably a consequence of an increased risk of thrombosis in the fine blood vessels in the germinal matrix region.

**Material and methods:**

The aim of this study was to evaluate the possible relationship between Factor V (FV) *1691G>A*, Factor II (FII) *20210G>A* mutations and methylenetetrahydrofolate reductase (MTHFR) *677C>T*; *1298A>C* and Factor XIII (FXIII) *103G>T* gene polymorphisms and the occurrence of IVH in 100 infants born from 24 + 0 to 32 + 0 weeks of gestation, born from singleton pregnancy, before 32 + 0 weeks of gestation, exposed to antenatal steroid therapy, and without congenital abnormalities.

**Results:**

IVH developed 45 (45%) infants, including 15 (33.33%) diagnosed with IVH stage I, 20 (42.22%) with stage II, 8 (17.77%) with stage III, and 3 (6.66%) with stage IV. Analysis showed a prevalence 4.5 times higher of IVH stages II to IV in infants with the genotype *CC* (OR 4511 (1147–17.75); *p* = 0.026) of MTHFR *1298A>C* gene polymorphism. Our investigation did not confirm any significant prevalence of IVH development in other studied mutations/polymorphisms.

**Conclusions:**

This study confirmed that the MTHFR *1298A>C* polymorphism is associated with the risk of IVH. IVH is a significant problem for preterm infants. In addition to little progress in preventing IVH in preterm babies, substantial research that is focused on understanding the etiology, mechanism, and risk factors for IVH is imperative. In the era of personalized medicine, identification of genetic risk factors creates opportunities to generate preventative strategies.

**Electronic supplementary material:**

The online version of this article (doi:10.1007/s00381-017-3460-8) contains supplementary material, which is available to authorized users.

## Introduction

Congenital thrombophilia is a genetic predisposition for venous or arterial thrombosis. The resistance of factor V to the anti-coagulant action of activated protein C is the most prevalent type of congenital thrombophilia. In fact, in over 90% of patients, it is caused by a point mutation of the factor V (FV) gene at position 1691 (*1691G>A*). The other types include a mutation in factor II (FII) at position 20210 in the 3′ untranslated region of the gene (*20210G>A*) and a polymorphism in methylenetetrahydrofolate reductase (MTHFR) gene at position 677 (*677C>T*) and at 1298 (*1298A>C*) [1–3].

The role of genetic polymorphisms involved in the coagulation pathway of intraventricular hemorrhage (IVH) pathogenesis is probably a consequence of an increased risk of thrombosis in the fine blood vessels in the germinal matrix region. Increased blood pressure in germinal matrix vessels may lead to vessel wall rupture and to IVH. As indicated in literature, congenital thrombophilia is associated with an increased IVH risk among newborns, but it may also play a protective role [4–9]. Thus, the role of the gene mutations that are involved in coagulation pathway in the pathogenesis of IVH remains unclear. The heterogeneity, size, and ethnic diversity of previously studied infants are the most likely explanation for the differences in these findings.

The aim of this study was to evaluate the possible relationship between FV *1691G>A* (R506Q), FII *20210G>A* mutations and MTHFR *677C>T* (A222V); *1298A>C* (E429A) and *103G>T* FXIII gene polymorphisms and the occurrence of IVH in a population of newborns born from 24 + 0 to 32 + 0 weeks of gestation.

## Material and methods

### Study population

In order to guarantee a homogenous group of patients, we created the following inclusion criteria for the study: Caucasian origin; neonates born from 24 + 0 to 32 + 0 weeks of pregnancy; singleton pregnancy; newborns with completed antenatal steroid therapy (AST), and newborns without chromosomal abnormalities, without toxoplasmosis, other, rubella, cytomegalovirus, and herpes (TORCH) infections, and also without inborn errors of metabolism. Based on inclusion criteria, we enrolled 100 of 428 (23.4%) infants into the study population. These patients were all born from 24 + 0 to 32 + 0 weeks of gestation in Clinical Hospital of Gynecology and Obstetrics at the University of Medical Sciences in Poznań, Poland, and then admitted to Neonatal Intensive Care Unit at Department of Neonatology between June 1, 2014, and August 15, 2016.

### Clinical features

The following risk factors that may associate with the development of IVH were studied: gender, gestational age (GA; weeks), birth weight (BW, g), small for gestational age (SGA, defined as BW under 3th percentile), type of delivery (vaginal birth vs. cesarean section), birth asphyxia (defined as Apgar score less than 6 at 10 min and pH <7.0 or blood base excess (BE) <−15 mmol/l in cord blood), intrauterine infection (defined as positive culture in sterile originally accompanied by clinical symptoms), and thrombocytopenia (defined as platelet count less than 100,000 per microliter of blood found in first 7 days of life) in neonates.

### IVH diagnosis

IVH was diagnosed by routine cranial ultrasound, which was performed on the first, third, and seventh days of life using the cranial ultrasonographic scanner (10-MHz transducer, Prosound α7 Premier, Aloka). The Papille IVH classification was used in staging IVH [10], and the results of cranial ultrasound were confirmed by two independent neonatologists.

### Studied polymorphisms

Based on most common etiology of inherited thrombophilia in Caucasian population, we studied the following gene: FV *1691G>A* (R506Q), FII *20210G>A*, and polymorphism of MTHFR *677C>T* (A222V) and *1298A>C* (E429A) and FXIII *103G>T* genes.

Samples of blood were taken after delivery, collected in EDTA, and banked. Genomic DNA was extracted from blood leukocytes using QIAamp DNA Blood Mini Kit (QIAGEN Inc., Germany) according to the manufacturer’s recommendations. Genotyping was performed using polymerase chain reaction-restriction fragment length polymorphism (PCR-RFLP) procedures. The description of the polymorphisms/mutations is shown in Table 1 [11–15].

**Table 1:** The description of the studied polymorphism genes

Polymorphism	The position of the gene on chromosome	Sequence of primers	Restriction enzyme	Identified sequence	Size of PCR product (bp)	Products
*MTHFR 677C>T (*rs1801133) [11]	1p36.3	5′ TGA AGG AGA AGG TGT CTG CGG GA 3′ 5′ AGG ACG GTG CGG TGA GAG TG 3′	*Hinf*I (Eurx)	G^ANTC	198	*CC*—198 bp; *CT*—198, 175, 23 bp; *TT*—175, 23 bp
*MTHFR 1298A>C* (rs1801131) [11]	1p36.3	5′ CTT CTA CCT GAA GAG CAA GTC 3′ 5′ CAT GTC CAC AGC ATG GAG-3′	*Mbo*II (Eurx)	GAAGA (8/7)	256	*AA*—176, 30, 28, 22 bp; *AC*—204, 30, 28, 22 bp; *CC*—204, 30, 22 bp
*FV 1691G>A* (rs6025) [12]	1q23	5′ TGC CCA GTG CTT AAC AAG ACC A 3′ 5′ CTT GAA GGA AAT GCC CCA TTA 3′	*Mnl*I (Eurx)	CCTC (7/6)	220	*GG*—116, 67, 37 bp; *GA*—153, 116, 67, 37 bp; *AA*—153, 67 bp
*FII 20210G>A* (rs3136516) [13]	11p11-q12	5′ TCT AGA AAC AGT TGC CTG GC 3′ 5′ ATA GCA CTG GGA GCA TTG AAG C3′	*Hind*III (Thermo Scientific)	A^AGCTT	345	*GG*—345 bp; *GA*—345, 322, 23 bp; *AA*—322, 23 bp
*FXIII 103G>T* (rs5985) [14]	6p25.1	5′ CATGCCTTTTCTGTTGTCTTC3′ 5′ ACCTTGCAGGTTGACGCCCCGGGGCACTA3′	*HpyF3I* (DdeI) (Thermo Scientific	C^TNAG	192	GG—192 bp; GT—192, 161, 31 bp; TT—161, 31 bp

Informed consent was obtained from parents of all infants enrolled into study.

The study followed the tenets of the Declaration of Helsinki and was approved by the Bioethics Committee of Poznan University of Medical Sciences (66/14 and 799/16). Furthermore, all methods and examinations were performed in accordance with the relevant ethical guidelines and regulations.

### Statistical analysis

The results are presented as percentage for categorical variables, or median (range) for non-normally distributed continuous variables as tested by the Shapiro–Wilk test. The Fisher exact probability test, the chi-squared test, Fisher–Freeman–Halton, and chi-squared test with Yates correction were used to evaluate the association between IVH and analyzed variables. Differences in non-normally distributed continuous variables were compared by the Mann–Whitney *U* test. The genotype and allele frequencies were compared between two groups: group 1: patients with or without IVH, with/without IVH grade I, and with IVH grades II–IV in the entire study group and group 2: with or without IVH, with/without IVH grade I, and with IVH grades II–IV in infants born from 24 + 0 to 28 + 0 weeks of gestation. A *p* value less than 0.05 is statistically significant. The expected genotype frequencies were calculated from allele frequencies with the Hardy-Weinberg equation. Statistical analysis was performed using Cytel Studio version 10.0, created January 16, 2013 (Cytel Studio Software Corporation, Cambridge, MA, USA), and Statistica version 10, 2011 (Stat Soft, Inc., Tulsa, OK, USA).

## Results

IVH developed 45 (45%) infants, including 15 (33.33%) diagnosed with IVH stage I, 20 (42.22%) with stage II, 8 (17.77%) with stage III, and 3 (6.66%) with stage IV.

No significant differences in the incidence of IVH were found between female (20; 44.44%) and male (25; 55.56%) neonates. The incidence of IVH stages II to IV was higher incidence with a lower GA, significantly higher in children born from 24 + 0 to 28 + 6 weeks of gestation compared to those born from 29 + 0 to 32 + 0 weeks of gestation (74.19 vs 25.81%; *p* = 0.007); higher incidence of a lower Apgar score in the first (6(1–10) vs 8(2–10); *p* = 0.007) and fifth minutes of life (4(1–10) vs 7(1–8); *p* = 0.001); and more often in children diagnosed with intrauterine infection (70.97 vs 47.83%; *p* = 0.031) and thrombocytopenia (45.16 vs 17.39%; *p* = 0.034). In the study population, 10 of 100 (10%) patients died. All children that died were born from 24 + 0 to 28 + 6 weeks of gestation (18.18%), 7 of which (70%) were diagnosed with IVH stages II to IV. Table 2 shows the characteristic of enrolled infants.

**Table 2:** Demographic and clinical characteristic of enrolled infants

	Group without IVH and IVH stage I	Group with IVH stage II- IV	*P* value
	*N* = 69 (%)	*N* = 31 (%)	
Gender			0.585
Male	36 (52.17)	18 (58.06)	
Female	33 (47.83)	13 (41.94)	
Gestational age (weeks)			0.007
24 + 0–28 + 6	31 (44.93)	23 (74.19)	
29 + 0–32 + 0	38 (55.07)	8 (25.81)	
Birth weight (g)			0.004
<750	6 (8.70)	8 (25.81)	
750–1000	15 (21.74)	12 (38.71)	
*>*1000	48 (69.57)	11 (35.48)	
Apgar score (median and range)			
1st minute	8 (2–10)	6 (1–10)	0.007
5th minute	7 (1–8)	4 (1–10)	0.001
Mode of delivery			0.148
Vaginal	25 (36.23)	16 (51.61)	
Cesarean section	44 (63.76)	15 (48.38)	
Asphyxia (pH lower than 7.0 or BE lower than −12)			0.468
Yes	1 (1.44)	2 (6.45)	
No	68 (98.56)	29 (93.55)	
Intrauterine infection			0.031
Yes	33 (47.83)	22 (70.97)	
No	36 (52.17)	9 (29.03)	
Thrombocytopenia			0.034
Yes	12 (17.39)	14 (45.16)	
No	57 (82.61)	17 (54.84)	
Deaths	3 (4.41)	7 (22.58)	0.015

Analysis showed a prevalence 4.5 times higher of IVH stages II to IV in infants with the genotype *CC* (OR 4511 (1147–17.75); *p* = 0.026) of MTHFR *1298A>C* gene polymorphism. There was a higher prevalence of allele *C* carriers of MTHFR *1298A>C* in patients with stage II to IV IVH (OR 1.816 (0.984–3.352); *p* = 0.056). Our investigation did not confirm any significant prevalence of IVH development in other studied mutations/polymorphisms. Genotype distribution of the studied mutations/polymorphisms in infants with/without IVH or with/without IVH grade I and with IVH grades II–IV is presented in Tables 3 and 4.

**Table 3:** Genotype distribution in infants without and with IVH or without/with IVH grade I and with IVH grades II–IV (*N* observed, *Exp* expected—genotype frequencies calculated from allele frequencies with the Hardy-Weinberg (H-W) equation)

Gene symbol		Group without IVH	Exp	Group with IVH grades I–IV	Exp	*P* value	OR	Group without IVH and IVH I	Exp	Group with IVH grades II–IV	Exp	*P* value	OR
		*N* = 54 (%)		*N* = 46 (%)				*N* = 69 (%)		*N* = 31 (%)			
FV *1691G>A*	Genotype												
	*GG*	49 (90.74)	49.12	44 (95.65)	44.02	–	References	63 (91.30)	63.13	30 (96.77)	30.01	–	References
	*GA*	5 (9.26)	4.77	2 (4.35)	1.96	0.579	0.446 (0.041–2.912)	6 (8.70)	5.74	1 (3.23)	0.98	0.599	0.35 (0.007–3.114)
	*AA*	0 (0.00)	0.12	0 (0.00)	0.02	–	–	0 (0.00)	0.13	0 (0.00)	0.01	–	–
	H-W		0.938		0.989				0.931		0.996		
	Allele												
	*G*	103 (95.37)		90 (97.83)		–	References	132 (95.65)		61 (98.39)		–	References
	*A*	5 (4.63)		2 (2.17)		0.587	0.458 (0.043–2.89)	6 (4.35)		1 (1.61)		0.608	0.361 (0.008–3.085)
FII *20210G>A*	Genotype												
	*GG*	54 (100.0)	54.00	46 (100.0)	46.00	–	–	69 (100.0)	69.00	31 (100.0)	31.00	–	–
	*GA*	0	0.00	0	0.00	–	–	0	0.00	0	0.00	–	–
	*AA*	0	0.00	0	0.00	–	–	0	0.00	0	0.00	–	–
	H-W		–		–				–		–		
	Allele			–									
	*G*	108 (100.0)		92 (100.0)		–	–	138 (100.0)		62 (100.0)		–	–
	*A*	0		0		–	–	0		0		–	–
MTHFR *677C>T*	Genotype												
	*CC*	30 (55.56)	27.45	24 (52.17)	25.88	–	References	39 (56.52)	36.96	15 (48.39)	16.33	–	References
	*CT*	17 (31.48)	22.10	21 (45.65)	17.25	0.418	1.544 (0.618–3.872)	23 (33.33)	27.08	15 (48.39)	12.34	0.341	1.696 (0.638–4.485)
	*TT*	7 (12.96)	4.45	1 (2.17)	2.88	0.175	0.179 (0.004–1.582)	7 (10.14)	4.96	1 (3.23)	2.33	0.661	0.371 (0.008–3.357)
	H-W		0.237		0.337				0.457		0.486		
	Allele												
	*C*	77 (71.30)		69 (75.00)		–	References	101 (73.19)		45 (72.58)		–	References
	*T*	31 (28.70)		23 (25.00)		0.670	0.828 (0.418–1.626)	37 (26.81)		17 (27.42)		1.000	1.031 (0.490–2.112)
MTHFR *1298A>C*	Genotype												
	*AA*	24 (44.44)	24.67	14 (30.43)	15.27	–	References	29 (42.03)	31.34	9 (29.03)	8.78	–	References
	*AC*	25 (46.30)	23.66	25 (54.35)	22.47	0.311	1.714 (0.667–4.454)	35 (50.72)	30.33	15 (48.39)	15.44	0.680	1.381 (0.480–4.128)
	*CC*	5 (9.26)	5.67	7 (15.22)	8.27	0.327	2.4 (0.528–11.4)	5 (7.25)	7.34	7 (22.58)	6.78	0.026	4.511 (1.147–17.75)
	H-W		0.917		0.747				0.441		0.988		
	Allele												
	*A*	73 (67.59)		53 (57.61)		–	References	93 (67.39)		33 (53.23)		–	References
	*C*	35 (32.41)		39 (42.39)		0.190	1.535 (0.827–2.848)	45 (32.61)		29 (46.77)		0.056	1.816 (0.984–3.352)
FXIII *103G>T*	Genotype												
	*GG*	28 (51.85)	28.17	21 (45.65)	20.89	–	References	35 (50.72)	35.51	14 (45.16)	13.56	–	References
	*GT*	22 (40.74)	21.67	20 (43.48)	20.22	0.807	1.212 (0.488–3.01)	29 (42.03)	27.98	13 (41.94)	13.89	0.984	1.121 (0.411–3.036)
	*TT*	4 (7.41)	4.17	5 (10.87)	4.89	0.730	1.667 (0.313–9.411)	5 (7.25)	5.51	4 (12.90)	3.56	0.565	2.00 (0.340–10.75)
	H-W		0.994		0.997				0.955		0.939		
	Allele												
	*G*	78 (72.22)		62 (67.39)		–	References	99 (71.74)		41 (66.13)		–	References
	*T*	30 (27.78)		30 (32.61)		0.556	1.258 (0.656–2.412)	39 (28.26)		21 (33.87)		0.523	1.3 (0.644–2.584)

**Table 4:** Genotype distribution in infants 24–28 weeks of gestation without and with IVH or without/with IVH grade I and with IVH grades II–IV (*N* observed, *Exp* expected—genotype frequencies calculated from allele frequencies with the Hardy-Weinberg (H-W) equation)

Gene symbol		Group without IVH	Exp	Group with IVH grades I–IV	Exp	*P* value	OR	Group without IVH and IVH I	Exp	Group with IVH grades II–IV	Exp	*P* value	OR
		*N* = 19 (%)		*N* = 35 (%)				*N* = 31 (%)		*N* = 23 (%)			
FV *1691G>A*	Genotype												
	*GG*	18 (94.74)	18.01	33 (94.29)	33.03	–	References	29 (93.55)	29.03	22 (95.65)	22.01	–	References
	*GA*	1 (5.26)	0.97	2 (5.71)	1.94	1.000	1.091 (0.053–67.89)	2 (6.45)	1.94	1 (4.35)	0.98	1.000	0.659 (0.011–13.53)
	*AA*	0 (0.00)	0.01	0 (0.00)	0.03	–	–	0 (0.00)	0.03	0 (0.00)	0.01	–	–
	H-W		0.993		0.985				0.983		0.994		
	Allele												
	*G*	37 (97.37)		68 (97.14)		–	References	60 (96.77)		45 (97.83)		–	References
	*A*	1 (2.63)		2 (2.86)		1.000	1.088 (0.055–65.91)	2 (3.23)		1 (2.17)		1.000	0.667 (0.011–13.23)
FII *20210G>A*	Genotype												
	*GG*	19 (100)	19.00	35 (100)	35.00			31 (100)	31.00	23 (100)	23.00		
	*GA*	0	0.00	0	0.00	–	–	0	0.00	0	0.00	–	–
	*AA*	0	0.00	0	0.00	–	–	0	0.00	0	0.00	–	–
	H-W		–		–				–		–		
	Allele												
	*G*	38 (100)		70 (100)				62 (100)		46 (100)			
	*A*	0		0		–	–	0		0		–	–
MTHFR *677C>T*	Genotype												
	*CC*	10 (52.63)	9.59	18 (51.43)	19.31	–	References	18 (58.06)	17.81	10 (43.48)	11.13	–	References
	*CT*	7 (36.84)	7.82	16 (45.71)	13.37	0.924	1.27 (0.338–4.933)	11 (35.48)	11.37	12 (52.17)	9.74	0.370	1.964 (0.554–7.017)
	*TT*	2 (10.53)	1.59	1 (2.86)	2.31	0.656	0.278 (0.004–6.222)	2 (6.45)	1.81	1 (4.35)	2.13	1.000	0.9 (0.014–19.45)
	H-W		0.902		0.508				0.984		0.538		
	Allele												
	*C*	27 (71.05)		52 (74.29)		–	References	47 (75.81)		32 (69.57)		–	References
	*T*	11 (28.95)		18 (25.71)		0.885	0.850 (0.325–2.298)	15 (24.19)		14 (30.43)		0.612	1.371 (0.531–3.515)
MTHFR *1298A>C*	Genotype												
	*AA*	5 (26.32)	6.96	11 (31.43)	12.01	–	References	10 (32.26)	12.90	6 (26.09)	6.26	–	References
	*AC*	13 (68.42)	9.08	19 (54.29)	16.99	0.759	0.664 (0.146–2.744)	20 (64.52)	14.19	12 (52.17)	11.48	1.000	1.000 (0.248–4.263)
	*CC*	1 (5.26)	2.96	5 (14.29)	6.01	0.917	2.273 (0.168–128.7)	1 (3.23)	3.9	5 (21.74)	5.26	0.149	8.333 (0.627–432.6)
	H-W		0.170		0.782				0.075		0.976		
	Allele												
	*A*	23 (60.53)		41 (58.57)		–	References	40 (64.52)		24 (52.17)		–	References
	*C*	15 (39.47)		29 (41.43)		1.000	1.085 (0.451–2.645)	22 (35.48)		22 (47.83)		0.275	1.667 (0.712–3.899)
FXIII *103G>T*	Genotype												
	*GG*	12 (63.16)	10.32	16 (45.71)	16.46	–	References	17 (54.84)	15.61	11 (47.83)	11.13	–	References
	*GT*	4 (21.05)	7.37	16 (45.71)	15.09	0.176	3.00 (0.691–15.22)	10 (32.26)	12.77	10 (43.48)	9.74	0.657	1.545 (0.416–5.74)
	*TT*	3 (15.79)	1.32	3 (8.57)	3.46	1.000	0.75 (0.055–6.683)	4 (12.90)	2.61	2 (8.7)	2.13	1.000	0.773 (0.061–6.568)
	H-W		0.137		0.938				0.481		0.992		
	Allele												
	*G*	28 (73.68)		48 (65.75)		–	References	44 (70.97)		32 (69.57)		–	References
	*T*	10 (26.32)		25 (34.25)		0.527	1.458 (0.570–3.912)	18 (29.03)		14 (30.43)		1.000	1.069 (0.424–2.661)

Seven patients needed ventriculo-peritoneal shunt placement. We did not find any link between studied polymorphisms and necessity of surgical intervention.

## Discussion

In our study, we evaluated the possible association between genes involved in the coagulation pathway and the development of IVH, in a large study population of preterm infants born from 24 + 0 to 32 + 0 weeks of gestation with the exposure to AST. It is hypothesized that increased fibrinolytic activity and decreased levels of clotting factors may contribute to the severity of IVH.

The univariate analysis confirmed the previously reported association of IVH with younger GA, lower Apgar score in first and fifth minutes of life, intrauterine infection, and thrombocytopenia [16–19].

### *677C>T*; *1298A>C* MTHFR polymorphisms

MTHFR is an enzyme that catalyzes the reduction of 5,10-methylenetetrahydrofolate to 5-methyltetrahydrofolate, a substrate in remethylation of homocysteine to methionine. The MTHFR is code by the gene on chromosome 1 location p36.2. Polymorphism of MTHFR gene consists of cytosine replaced by thymine at position 677 or adenosine replaced by cytosine at position 1298 in the gene, encoding a thermolabile enzyme with reduced activity, consequently resulting in increased blood plasma homocysteine concentration [20–22]. Hyperhomocysteinemia may lead to injury of vascular endothelium and lead to stroke, thrombosis, migraine, and vascular disorder IVH [20, 22]. Aden et al. evaluated genotypes for seven genes from 224 inborn, preterm infants with BW 500–1250 g, treated with AST and grade III IVH. MTHFR *1298A>C* polymorphism was more prevalent in cases of IVH [8]. Based on our results in a comparable study population, we confirmed 4.5-fold increased prevalence of IVH stages II to IV in patients with the genotype *CC* of MTHFR *1298A>C* gene polymorphism. We did not find any statistical significance association of MTHFR *677C>T* polymorphism and IVH occurrence. Ment et al. demonstrated that MTHFR *1298A>C* gene polymorphism is an independent risk factor for IVH. MTHFR *677C>T* polymorphism increases the risk of IVH in patients with low Apgar score. Ment et al. studied different populations compared to us, including multiple pregnancies and infants with body weight 500–1250 g [9].

### *1691G>A* FV mutation

Activated protein C resistance (APCR) is the resistance of FV to the anti-coagulant action of APC. APCR is the most prevalent type of congenital thrombophilia, and in over 90% of patients, it is caused by a mutation of the FV gene on chromosome 1 (the Leiden mutation). Factor V (FV) is synthesized by hepatocytes, monocytes, macrophages, and megakaryocytes. FV undergoes thrombin-dependent activation and APC-dependent inactivation. FV is transformed to its active form (factor Va (FVa)) by the thrombin that cleaves FV at Arg709, Arg1018, and Arg1545 within the B domain of FV. FVa with FXa and calcium ions forms the FIIase complex that converts FII into thrombin. FVa plays also an anti-coagulant role (APC-dependent) by the proteolysis of FVa to FVi, when APC is attached to FVa at Arg306, Arg506, and Arg679 of the heavy chain of FVa. Connecting APC to FVa at Arg506 and forming FVac inactivates FVIIIa [2, 23–25]. FV Leiden mutation is an autosomal dominant genetic mutation and occurs between 2 and 10% of the Caucasian race and in 90% of cases is caused by the replacement of arginine at position 506 of the heavy chain with glutamine, which results in resistance to APC-dependent proteolysis and retained pro-coagulant activity of FV [2]. In our study population, mutated allele *A* was found in 7% patients (only heterozygotes *GA*). We evaluated a role of mutation Leiden in pathogenesis of IVH development. The results of previous studies are unclear. Gopel et al. indicated an association of mutation FV (Leiden) with the incidence of IVH grades 1 and 2 and with protection role of it against IVH progression and extension [4]. In contrast, Ryckman et al. showed that heterozygotes may be predisposed for IVH grade I and II occurrence, but not grades III and IV [7]. Ramenghi et al. indicated that the risk ratio for IVH was 2.65 higher in carriers of mutation FV Leiden. The presence of mutation was associated with the severity of IVH [26]. Komlosi et al. [6], Aden et al. [8], Baier et al. [27], Petaja et al. [28], and Aronis et al. [29] did not find any association mutation of gene FV (Leiden) with IVH in preterm newborns. We did not find any association between FV Leiden mutation and incidence of IVH in preterm infants. However, the lack of association detected must be interpreted with caution, due to our small sample size with mutated allele *A* (*n* = 7).

### *20210G>A* FII mutation

Factor II (FII) is a vitamin K-dependent pro-enzyme produced in the liver. The role of FII is converting fibrinogen to fibrin. The FII-encoding gene is located on chromosome 11 (region: p11–q12). The replacement of guanine at position 20210 with adenine is associated with higher levels of FII synthesis. It has been shown that the mutation of the FII gene is related to the incidence of thrombosis in certain venous locations (portal vein, intracranial veins). Being a carrier of both FV Leiden gene mutation and the FII G20210A mutation increases the risk of thrombotic incidents, and in the Polish population, carriers are estimated at approximately 1% [30]. We have not found any patient in our study population with a mutated allele *A*. That is the reason why further analysis of the FII G20210A mutation and its impact on IVH incidence in preterm infants was not performed. In contrast to previous studies published by Baier et al. [27], Gopel et al. [4], Hartemann et al. [31], Petaja et al. [28], Ryckman et al. [7], and Aden et al. [8], Ramenghi showed that infants with VLBW and heterozygous for FII G20210A mutation are at increased risk for developing IVH [26].

### *103G>T* factor XIII polymorphism

FXIII plays an important role in the terminal phase of the clotting cascade. FXIII is composed of A and B subunits. FXIII is activated by thrombin in the presence of calcium by dissociation of subunit B, which consequently stabilizes the fibrin clot and increases its resistance to fibrinolysis.

*103G>T* FXIII polymorphism is caused by a point mutation in codon 34 of exon 2 of the FXIII gene and leads to valine-leucine change in the subunit A of the FXIII. The *103G>T* polymorphism in subunit A is located in the activation peptide 3 amino acid, near the thrombin activation side [32]. The FXIII *103G>T* polymorphism accelerates activation of FXIII by thrombin and changes the structure of fibrin clots into thinner fibers that are more densely packed [33]. Homozygous and heterozygous carriers of the FXIII *103G>T* polymorphism have higher rates of hemorrhagic stroke among adults [34], but not in children [35].

Gopel et al. showed that very-low-body-weight infants who carried the factor XIII 34Leu allele had a moderately increased risk of IVH development [36]. These findings were not confirmed by Ryckman et al. [7]. In our study, we did not confirm any link between IVH occurrence and polymorphism FXIII *103G>T*.

## Conclusions

This study confirmed that the MTHFR *1298A>C* polymorphism is associated with the risk of IVH. IVH is a significant problem for preterm infants. In addition to little progress in preventing IVH in preterm babies, substantial research that is focused on understanding the etiology, mechanism, and risk factors for IVH is imperative. In the era of personalized medicine, identification of genetic risk factors creates opportunities to generate preventative strategies.

## Electronic supplementary material

ESM 1 (XLS 42 kb)
